# Spatial Differentiation and Environment-Driven Mechanisms of Locust Community Structure in the Xinjiang Region Along the Sino-Kazakh Border

**DOI:** 10.3390/insects17030348

**Published:** 2026-03-22

**Authors:** Siqi Lin, Yongjun Zhang, Yating Guo, Huixia Liu, Jun Lin, Rong Ji, Roman Jashenko, Lan He

**Affiliations:** 1 College of Life Sciences, Xinjiang Normal University, International Research Center of Cross-Border Pest Management in Central Asia, Xinjiang Key Laboratory of Special Species Conservation and Regulatory Biology, Urumqi 830017, China; 17590931780@163.com (S.L.); heie@sina.com (Y.Z.); guoytchn@163.com (Y.G.); xjlhx0217@163.com (H.L.); jirong@xjnu.edu.cn (R.J.); 2 Tacheng, Research Field (Migratory Biology), Observation and Research Station of Xinjiang, Tacheng 834700, China; 3 Xinjiang Uygur Autonomous Region Grassland Biological Disaster Prevention and Control Center, Urumqi 830017, China; xjcy2009@163.com; 4 Changji University, Changji 831100, China; 5 Institute of Zoology, Ministry of Education and Science of Kazakhstan, Almaty 050038, Kazakhstan; rjashenko@gmail.com

**Keywords:** locust community, diversity, environmental driving factors, Sino-Kazakh border, grassland type

## Abstract

This study was conducted in the Xinjiang region, China, along the Sino-Kazakh border, an area recognized as high-risk for locust outbreaks and characterized by ongoing shifts in dominant pest species. This study systematically examined the structural characteristics of locust communities across different grassland types and identified the underlying environmental driving mechanisms. Field surveys were conducted to assess the diversity characteristics, density variations, and niche width of the locust communities across the different grassland types. The locust community in the mountain meadows had a significantly lower Shannon diversity index compared with the other grassland types (*p* < 0.05). Although the Simpson dominance index and Pielou evenness index were also the lowest in the mountain meadows, these differences were not statistically significant (*p* > 0.05). Permutational multivariate analysis of variance (PermANOVA) revealed highly significant differences in locust density among the grassland types (*p* = 0.001). Ecological niche analysis revealed stronger interspecific competition in the lowland meadow, and weaker competition in the temperate steppe-enriched deserts and temperate desert grasslands. Structural equation modeling and random forest analysis identified soil organic, plant total potassium, and soil pH as the key factors driving locust community structure across grassland types. This study clarifies the diversity patterns of locust communities in the Xinjiang region along the Sino-Kazakh border and provides empirical data to better understand locust community structure and distribution. It also offers a scientific basis for developing sustainable locust management strategies.

## Simple Summary

This study examined locust communities in the high-risk outbreak region along the Sino-Kazakh border in Xinjiang, China, to understand their structural variation across grassland types and the environmental drivers of these patterns. Field surveys revealed significant differences in locust diversity and density between grassland types, with mountain meadows showing the lowest locust diversity. Interspecific competition was strongest in the lowland meadows and weakest in the temperate desert steppes. Analyses identified soil organic matter, plant total potassium, and soil pH as the key environmental drivers shaping locust community structure. These findings clarify the distribution patterns of locusts in this region, providing essential data for understanding their ecology and for developing sustainable management strategies.

## 1. Introduction

Grassland ecosystems are the predominant ecosystem type in arid and semi-arid regions, and as critical ecological barriers, are essential for livestock production. They play an indispensable role in maintaining regional ecological security, regulating the climate, and conserving biodiversity [1,2,3,4]. However, due to significant hydrothermal constraints, arid and semi-arid ecosystems exhibit heightened sensitivity and vulnerability to global climate change and anthropogenic disturbances (including overgrazing and land-use changes). Thus, arid and semi-arid grassland ecosystems are a critical focal region for global change ecology research [5].

Insects are fundamental components of grassland ecosystems and help sustain critical ecosystem services due to their high species diversity and multi-functional ecological roles across trophic levels. Insects are also highly sensitive to environmental perturbations within their habitats [6] and are thus ideal bio-indicators for investigating interspecific distribution patterns and community-level divergences across habitat types [7]. Within European grassland ecosystems, Orthopterans are effective bioindicators of land-use change and habitat succession, showing rapid and distinct responses to management practices such as grazing and abandonment [8]. However, in the ecologically distinct arid and semi-arid regions of Asia, relevant research remains relatively scarce. Insects adapt to specific habitats via physiological regulatory mechanisms and spatial niche partitioning along environmental gradients [9]. Additionally, there are pronounced taxon-specific differences in insect responses to habitat modification [10]. This differential response pattern reflects species-specific ecological adaptations with cascading effects on the structural integrity and functional resilience of grassland ecosystems through trophic-level asymmetry. Consequently, exploring the interaction between the habitat and insect communities has significant theoretical and practical implications for the conservation of biodiversity and the preservation of ecosystem stability [11].

The spatial distribution patterns of insect communities reflect fundamental ecological processes, including species coexistence, resource competition, and environmental adaptation. Temperature, as a fundamental abiotic environmental factor, exerts a direct regulatory control over insect populations through modulation of critical physiological parameters, including developmental rate thresholds, metabolic scaling of growth trajectories, and reproductive fitness optima. These factors collectively affect population dynamics across thermal gradients [5]. Alterations in environmental parameters, such as temperature and humidity, not only significantly alter the species composition of insect communities but can also drive poleward range shifts in the northern hemisphere [12,13]. Long-term studies on grassland Orthopteran communities in Europe have shown that the relative impacts of land-use and climate change on grasshopper range shifts vary over different historical periods [14]. They also demonstrated that habitat availability and climate warming are primary drivers of distributional changes in grassland grasshoppers [15]. For arid and semi-arid ecosystems, changing precipitation regimes can restructure the plant community composition and productivity [16], triggering cascading effects on insect diversity via moisture-mediated pathways [17]. Moreover, plant diversity and soil properties within insect habitats directly and indirectly influence insect diversity. For instance, significant positive linear correlations have been recorded between beetle species richness and diversity indices and soil moisture and organic matter content in desert ecosystems [18]. In addition, Poniatowski et al. demonstrated that higher plant species complexity is associated with greater diversity in locust communities [19].

Over the past decade, the spatial distribution patterns of pest populations and their ecological regulation mechanisms have received increasing attention in integrated management research [20]. As a key functional group and potential disaster-causing species in grassland ecosystems, understanding the community structural characteristics of locusts and their driving mechanisms could reveal the function-maintaining mechanisms in arid and semi-arid regions. Furthermore, it could inform biodiversity conservation strategies, which could be used to optimize sustainable utilization patterns of grasslands and adaptive pest management in the context of global climate change.

The border region between China and Kazakhstan represents a typical arid to semi-arid ecosystem encompassing diverse grassland types, including desert grasslands, montane grasslands, and meadows. This area experiences complex climatic patterns influenced by the interaction between the westerly wind circulation and the Mongolian high-pressure system. Previous studies have demonstrated that the region provides favorable ecological conditions for locust species, including *Calliptamus italicus* (Linnaeus, 1758) and *Locusta migratoria migratoria* (Linnaeus, 1758) [21]. Yu et al. analyzed the breeding habitats and migration trajectories of locusts along the Sino-Kazakh border, identifying nine distinct migration routes through trajectory modeling [22]. In addition, Zha et al. employed the Weather Research and Forecasting mesoscale model to simulate synoptic conditions during migratory locust landings in the Tacheng region to elucidate the meteorological mechanisms governing locust descent along the Sino-Kazakh border [23]. These studies showed that global climate change and intensifying human activities may increase the future risk of locust outbreaks in this region, primarily due to its unique transitional zone properties and fragile ecosystem [24]. Although studies have recorded changes in both the dominant locust species and their distribution characteristics in Xinjiang, China [25], research on the distribution patterns of locust communities and their driving mechanisms in the Sino-Kazakh border region is limited. A comprehensive investigation into the distribution patterns and ecological niche characteristics of locust communities in the region will provide data for ecological niche theory assembly in arid zones. It will also establish a foundation for locust monitoring and early warning and green control systems as part of the Belt and Road initiative.

This study investigated the community structure and ecological niche characteristics of locust populations in the Sino-Kazakh border region. Random Forest (RF) and piecewise Structural Equation Modeling (piecewiseSEM) assessed how environmental factors—including climatic variables, altitude, and physicochemical properties of the plants and soils—influence the locust community. It aimed to identify the predominant factors governing locust spatial distribution to provide a basis for predicting potential locust distributions across the Sino-Kazakh border region. Importantly, data can aid in establishing a theoretical foundation to support early-warning systems for locust outbreaks. Accordingly, the following hypotheses were proposed: (1) differences in soil properties, moisture conditions, and plant composition across distinct grassland types drive the formation of diverse microhabitats and shape the locust community structure via ecological niche differentiation (e.g., spatial resource utilization); (2) the relative influence of ecological factors on locust community structure varies, and large-scale environmental factors (e.g., temperature and precipitation) exert their influence through multi-level cascading effects.

## 2. Materials and Methods

### 2.1. Study Area

The study area encompassed Xinjiang (China) along the Sino-Kazakh border, specifically covering four prefectures: Altay, Ili, Tacheng, and Bortala Mongol Autonomous Prefecture in northwestern China. The study area has a continental arid and semi-arid climate, characterized by complex topography and diverse grassland types. Xinjiang Grassland Resources and Their Utilization was used to classify the grassland types in the study area into five categories: lowland meadow, mountain meadow, temperate steppe, temperate desert-steppe, and temperate desert grassland [26].

### 2.2. Surveys

#### 2.2.1. Locust Communities

**Figure 1:**
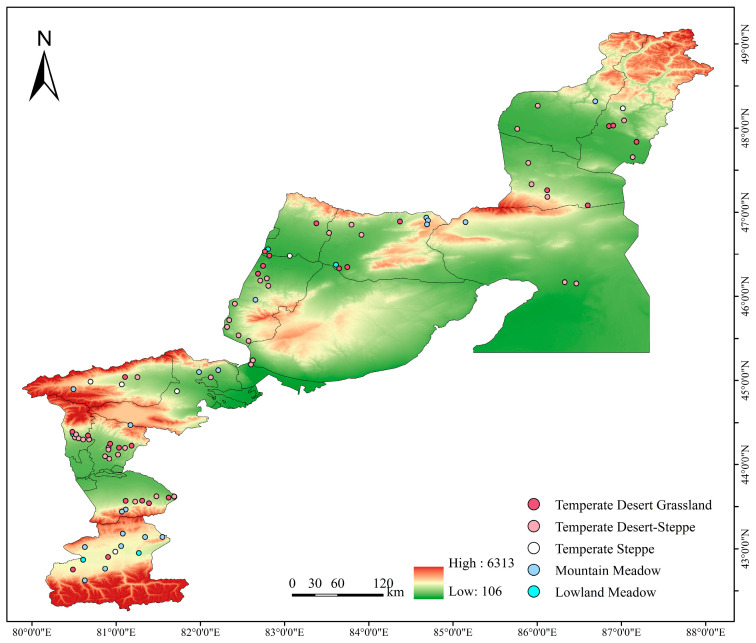
Overview Map of the Study Area. Note: The map displays the spatial distribution of all sampling points, with color-coded dots designating distinct grassland types. The base maps were sourced from the Standard Map Service website of China’s National Bureau of Surveying, Mapping and Geoinformation (approval no. GS(2024)0650) and remain unaltered.

Field data were collected from late June to mid-July 2024, with surveys scheduled to avoid extreme weather conditions. A total of 94 sampling plots (50 m × 50 m) were established with three parallel transects per plot spaced 10 m apart (Figure 1). Standardized sweep-netting was conducted (180° bilateral arc swings), with 50 sweeps per transect, generating 150 sweeps per plot and 14,100 sweeps in total. Locust species and abundances were documented in situ. Unidentified specimens were labeled with site data, preserved in ethyl acetate killing jars, and transported to the laboratory for integrated morphological and molecular species identification. This was followed by quantitative population analysis. The sample plots’ latitude, longitude, and elevation were documented using Global Positioning System technology.

#### 2.2.2. Vegetation Community

Within each 50 × 50 m sampling plot, three 1 × 1 m vegetation sample quadrats were established at intervals of 30 m. Vegetation within these sample plots was investigated using the diagonal sampling method. The vegetation species, height (in cm), cover (in %), and fresh weight (in g) within each sample plot were measured. Subsequently, all the vegetation within each sample plot was cut flush to the ground, and the cuttings were transported to the laboratory. The dry weight and the carbon, nitrogen, phosphorus, potassium, and other nutrient contents were determined from these cuttings.

#### 2.2.3. Soil Sampling

Three soil samples were collected at a depth of 0–10 cm from each plot using a soil auger. After thorough mixing, 1000 g of the soil was used for analysis of the soil physicochemical properties. Bulk density cores were extracted from pedogenically defined horizons using a 100 cm^3^ cutting ring (inner diameter: 5.05 cm, height: 5.02 cm; ISO 11272:2017 compliant [27]) with a stratified sampling design ensuring representation of each genetic horizon.

#### 2.2.4. Plant Physicochemical Analysis

Plant samples were dried at 65 °C until a constant mass was reached (48 h), and the dry weights were documented. The leaf tissues were pulverized and passed through a 0.15 mm sieve. The organic carbon was quantified via potassium dichromate oxidation-external heating, the total nitrogen (TN) by perchloric-sulfuric acid digestion, the total phosphorus (TP) by acid dissolution-molybdenum-antimony anti-colorimetry, and the total potassium (TK) by ammonium acetate extraction-flame photometry [28,29,30].

#### 2.2.5. Soil Physicochemical Properties

To determine the gravimetric water content, the soil samples that were collected for bulk density analysis were oven-dried at 65 °C to a constant weight, ground, and passed through a 2 mm sieve to separate out the gravel, which was weighed along with the fine earth fraction. For the other physicochemical analyses, separate soil samples were air-dried and split into two subsamples. One subsample was sieved (<1 mm), and the pH, electrical conductivity, salinity, and total dissolved solids were measured. The other subsample was passed through a 0.25 mm sieve, and the soil organic carbon (SOC), TN, TP, and TK were measured. The SOC was determined using the potassium dichromate oxidation-external heating method. The TN was measured using the semi-micro Kjeldahl method. Both the TP and TK were determined after NaOH fusion, with the TP quantified by the molybdenum-antimony colorimetric method and the TK by flame photometry [31].

#### 2.2.6. Ecological Factor Data Sources

Meteorological data were obtained from the ERA5-Land reanalysis data, facilitated by the Copernicus Climate Change Service (C3S, http://climate.copernicus.eu/, accessed on 7 May 2025) of the European Centre for Medium-Range Weather Forecasts. The data were imported into ArcGIS Desktop 10.8 [32], and the Multi-Value Extraction to Points tool was used to extract meteorological data corresponding to the surveyed sample plots.

### 2.3. Data Analysis

#### 2.3.1. Dominance Classification of Locusts

Based on the relative abundance of the individual species within each taxonomic unit compared with the total number of species, locusts were classified into three major categories: dominant species (abundance > 10%), common species (1% < abundance ≤ 10%), and rare species (abundance ≤ 1%) [33].

#### 2.3.2. Locust Diversity

The diversity of the locusts in the sample plots, including the diversity index, dominance index, and evenness index, was analyzed with the Shannon index (H), Simpson index (C), and Pielou index (E) [34,35,36].

The formulas for these indices are as follows:

Shannon Diversity Index:

(1) $$H^{\prime }=-\sum P_{i}\ln P_{i}$$

Simpson Dominance Index:

(2) $$D=1-\sum P_{i}$$

Pielou Evenness Index:

(3) $$J=H^{\prime }/\ln S$$

where *S* represents the total number of species in the quadrat. $P_{i}=N_{i}/N$, where $P_{i}$ is the relative abundance of the *i*-th species, $N_{i}$ is the number of individuals of the *i*-th species in the quadrat, and N is the total number of species recorded in the quadrat.

#### 2.3.3. Locust Community Structure in Different Grassland Types

The β-species diversity was used to assess differences in the species composition between the various ecosystems. It serves as a measure of the diversity across different habitats within a region, reflecting the variation in species composition of ecosystems. Based on the Bray–Curtis dissimilarity matrix, non-metric multidimensional scaling (NMDS) with two-dimensional and three-dimensional solutions was evaluated, and the stress value was calculated to assess the goodness-of-fit of the ordination. A Permutational multivariate ANOVA (PermANOVA) tested whether there were significant differences in community structure among the different groups. Both the NMDS and PermANOVA were implemented using the “vegan” and “ggpubr” packages in R 4.2.2 [37].

Analysis of similarities (ANOSIM) using the Jaccard dissimilarity index highlighted significant differences in grasshopper community composition among grassland types. It generates an R-statistic ranging from −1 to 1, where values close to 1 indicate strong separation between groups, values near 0 suggest no difference, and negative values imply greater dissimilarity within groups than between groups. Significance was assessed using 999 permutations. This analysis was conducted in R 4.2.2 with the “vegan”, “tidyverse”, and “ggplot2” packages.

The Jaccard similarity coefficient was selected to compare the similarity of the species in the different grassland types. This analysis was completed using the “vegan” and “ggplot2” packages in R 4.2.2 software.

The formula for this index is as follows:

Jaccard index:

(4) $$J(A,B)=c/\left(a+b\right)-c$$

where “*a*” represents the total number of species in set A, “*b*” represents the total number of species in set B, and “*c*” represents the number of species common to both sets.

#### 2.3.4. Niche Breadth and Niche Overlap

The niche breadth and niche overlap of the locusts were calculated based on the Levins index [38] and the Pianka index [39]. In this study, each sampling plot was considered an independent resource state. Therefore, for the Levins index, the niche breadth ranges from 1 to N, where N represents the total number of sampling plots within a given grassland habitat type. The formulas are as follows:

Levins index:

(5) $$B_{i}=\frac{1}{\sum_{j=1}^{r}{\left(P_{ij}\right)}^{2}}$$

Pianka Index:

(6) $$O_{jk}=\sum_{i=1}^{n}\frac{P_{ij}P_{ik}}{\sqrt{\sum_{i=1}^{n}{P_{ij}}^{2}\sum_{i=1}^{n}{P_{ik}}^{2}}}$$

#### 2.3.5. Structural Equation Model and Random Forest Model

PiecewiseSEM was used to establish theoretical relationships between variables, assess the reliability of the hypothesized model, and quantify both the direct and indirect effects between variables. Here, the latent variable “Locust community” was represented by the first principal component (PC1) derived from a principal component analysis (PCA) based on species abundance data.

To evaluate the relative importance of environmental factors in shaping grasshopper community structure (PC1), a random forest regression was performed. The model was constructed with 500 trees (ntree = 500), and all predictor variables were included. Variable importance was measured as the increase in mean squared error (%IncMSE) when a predictor was permuted. Permutation tests assessed the statistical significance of the model and individual predictors. Overall model significance was tested with 500 permutations (nperm = 500) and 1000 trees (ntree = 1000), which compares the observed R^2^ to a null distribution generated by randomly shuffling the response variable. Predictors with *p* < 0.05 were considered statistically significant. A variance inflation factor (VIF) analysis removed ecological factors with strong collinearity (VIF > 10).

In R version 4.2.2, PCA was performed with the “FactoMineR” and “factoextra” packages. The piecewiseSEM analysis was conducted using the “piecewiseSEM”, “nlme”, and “lme4” packages. The RF modeling was performed using the “randomForest”, “rfUtilities”, “rfPermute”, and “dplyr” packages. VIF analysis was performed with the “car” package.

## 3. Results

### 3.1. Compositional Differences in the Locust Communities Across Grassland Types

A total of 6036 locust individuals were captured during the survey period, belonging to 34 species, 23 genera, and four families. Table 1 lists the major locust species recorded from the different grassland types in the Xinjiang region along the Sino-Kazakh border and their relative dominance values. Overall, *Dociostaurus* spp., *Calliptamus italicus*, and *Calliptamus barbarus barbarus* (Costa, 1836) were identified as the dominant species. Common species included *Oedaleus decorus* (Germar, 1825), *Calliptamus coelesyriensis* Giglio-Tos, 1893, *Oedipoda miniata* (Pallas, 1771), *Stauroderus scalaris* (Fischer von Waldheim, 1846), *Omocestus haemorrhoidalis* (Charpentier, 1825), *Sphingonotus coerulipes* Uvarov, 1922, *Ramburiella turcomana* (Fischer von Waldheim, 1833), *Chorthippus biguttulus* (Linnaeus, 1758), and *Chorthippus albomarginatus* (De Geer, 1773), while the remaining species were considered rare.

In the lowland meadows (*n* = 5 plots), 14 locust species were recorded. *Calliptamus italicus*, *Dociostaurus* spp., *Ramburiella turcomana*, and *Oedaleus decorus* were the dominant species, whereas *Parapleurus alliaceus* (Germar, 1817), *Calliptamus barbarus*, *Oedipoda miniata*, *Oedipoda caerulescens* (Linnaeus, 1758), *Omocestus haemorrhoidalis*, and *Omocestus petraeus* (Brisout de Barneville, 1856) were common. The remaining species were rare.

The mountain meadows (*n* = 19 plots) hosted 20 species, with *Dociostaurus* spp., *Stauroderus scalaris*, and *Calliptamus italicus* as dominant species. Common species included *Omocestus haemorrhoidalis*, *Chorthippus biguttulus*, *Pararcyptera microptera* (Fischer von Waldheim, 1833), *Aeropus sibiricus* (Linnaeus, 1767), *Chorthippus albomarginatus*, *Bryodema gebleri* (Fischer von Waldheim, 1836), *Calliptamus coelesyriensis*, *Omocestus viridulus* (Linnaeus, 1758), *Oedaleus decorus*, and *Stenobothrus lineatus* (Panzer, 1796).

The temperate steppes (*n* = 6 plots) yielded 16 species. *Dociostaurus* spp., *Oedaleus decorus*, and *Calliptamus barbarus* were dominant. Common species were *Bryodema gebleri*, *Calliptamus italicus*, *Sphingonotus nebulosus* (Fischer von Waldheim, 1846), *Chorthippus biguttulus*, *Oedipoda caerulelescens*, *Sphingonotus coerulipes*, *Chorthippus albomarginatus*, *Oedipoda miniata*, *Stenobothrus lineatus*, *Notostaurus albicornis* (Eversmann, 1848), and *Omocestus haemorrhoidalis*, while *Calliptamus coelesyriensis* and *Stauroderus scalaris* were rare.

In the temperate desert-steppes (*n* = 39 plots), 22 species were collected. *Calliptamus italicus*, *Dociostaurus* spp., *Calliptamus barbarus*, and *Calliptamus coelesyriensis* were dominant. *Oedaleus decorus*, *Oedipoda miniata*, *Sphingonotus coerulipes*, and *Notostaurus albicornis* were common, and the rest were rare.

The temperate desert grasslands (*n* = 25 plots) also hosted 22 species, with *Calliptamus italicus*, *Dociostaurus* spp., and *Calliptamus barbarus* as dominant species. Common species included *Oedaleus decorus decorus*, *Oedipoda miniata*, *Calliptamus coelesyriensis*, *Omocestus haemorrhoidalis*, *Chorthippus albomarginatus*, and *Oedipoda caerulelescens*, while the other species were rare.

### 3.2. Analysis of Locust Community Diversity Between Grassland Types

**Figure 2:**
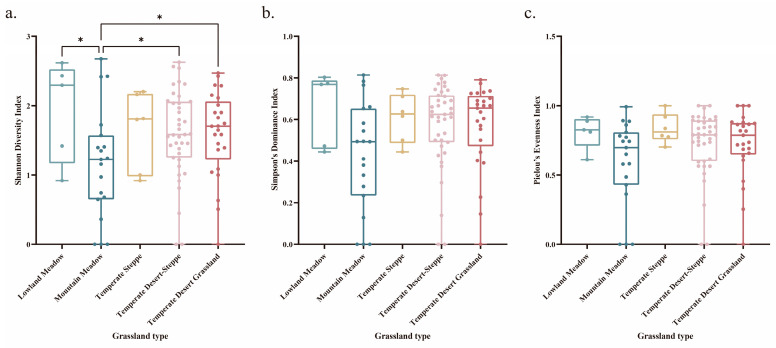
Diversity of locust communities across grassland types. Note: (**a**) Diversity indices, (**b**) Dominance index, and (**c**) Evenness index. An asterisk (*) indicates significant differences at *p* < 0.05.

Figure 2 illustrates variations in the locust diversity across the different grassland types. The Shannon–Wiener diversity index was lowest in the mountain meadows, which was significantly lower than that in the other grassland types (*p* < 0.05). Although the Simpson dominance index and Pielou evenness index were also lowest in the mountain meadows, there were no significant differences among the grassland types (*p* > 0.05).

### 3.3. Analysis of Differences and Similarities in the Locust Communities Between Grassland Types

**Figure 3:**
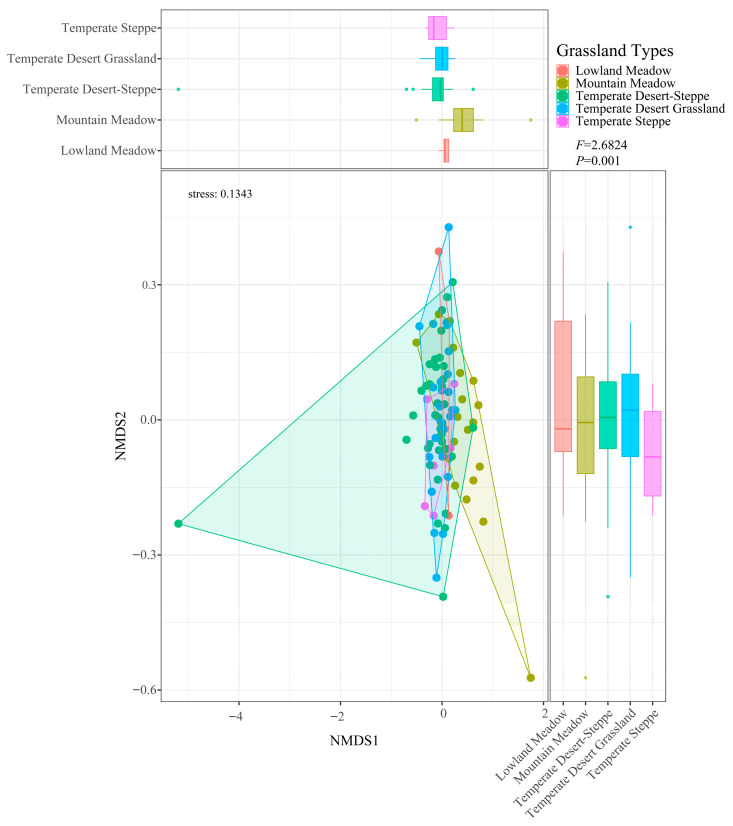
NMDS and PermANOVA Analyses of Grasshopper Communities in Different Grassland Types in the Sino-Kazakh Border Region.

**Figure 4:**
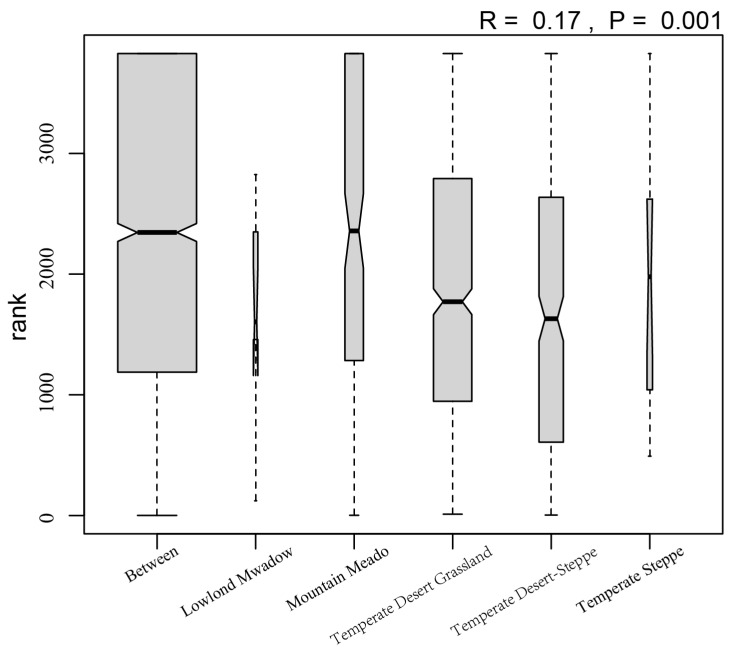
Boxplot of Jaccard similarity within and between the five grassland types.

The two-dimensional NMDS and PermANOVA results revealed highly significant differences in the locust community structure across the grassland types (F = 2.6824, *p* = 0.001, stress = 0.1343; Figure 3). To assess the robustness of the ordination, a three-dimensional NMDS solution was computed, which yielded a slightly lower stress value (stress = 0.1144; Figure S1), indicating an improved fit. The consistent patterns observed between the 2D and 3D solutions corroborate the reliability of the community differentiation presented in the main text. The ANOSIM analysis (Figure 4) revealed that within-group Jaccard similarity (0.124) was significantly higher than between-group similarity (0.093). Although the global R value was relatively modest (R = 0.17, *p* = 0.001), results indicate that grassland type plays a significant role in shaping grasshopper community composition (Table 2). The locust communities between temperate desert-steppe and temperate desert grassland exhibited the highest similarity, with a Jaccard index of 0.52, indicating high similarity. Moderate similarity was observed between lowland meadow and temperate steppe, as well as between mountain meadow and temperate desert grassland, with Jaccard indices of 0.34 and 0.29, respectively. In contrast, the locust communities between every other pair of grassland types showed low similarity, particularly between temperate desert-steppe and lowland meadow, mountain meadow, and temperate steppe, with Jaccard indices of 0.07, 0.15, and 0.05, respectively.

### 3.4. Analysis of the Niche Differences in the Locusts Among the Grassland Types

**Figure 5:**
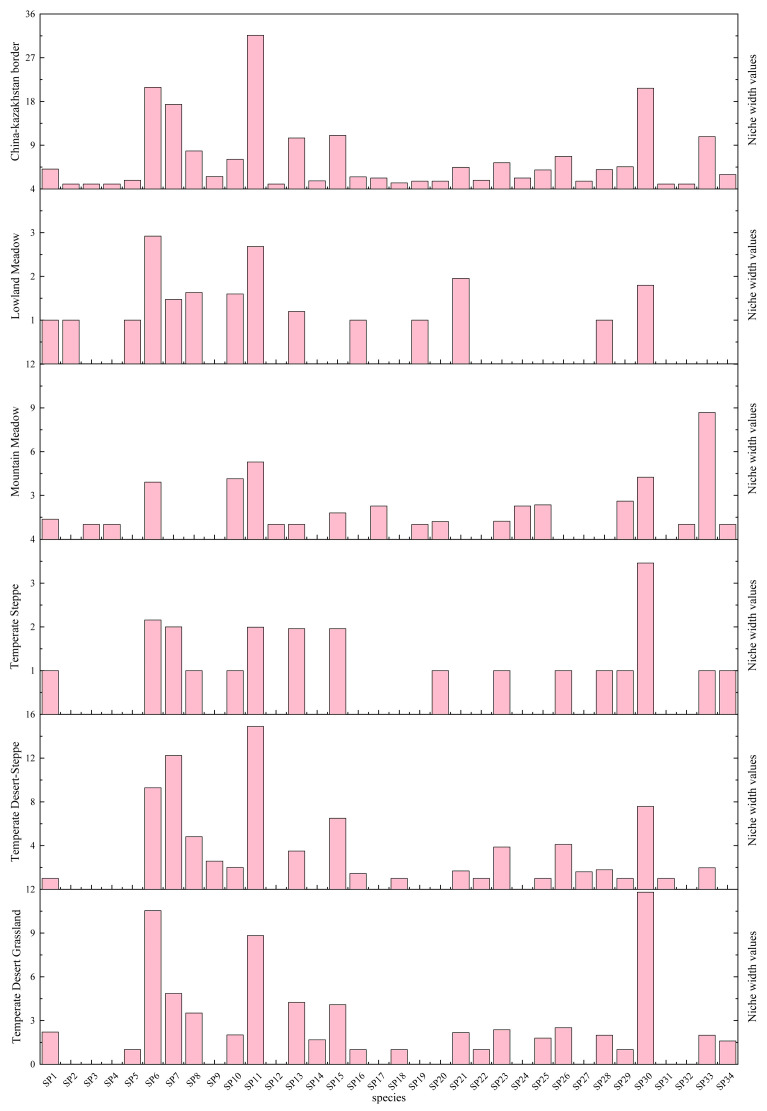
Niche breadth of locust species in the Sino-Kazakh border region and its major grassland types. Note: SP1: *Chorthippus albomarginatus*; SP2: *Parapleurus alliaceus*; SP3: *Euchorthippus pulvinatus*; SP4: *Chorthippus dichrous*; SP5: *Celes variabilis variabilis*; SP6: *Oedaleus decorus decorus*; SP7: *Calliptamus barbarus barbarous*; SP8: *Oedipoda miniata*; SP9: *Dericorys annulata roseipennis*; SP10: *Omocestus haemorrhoidalis*; SP11: *Dociostaurs* spp.; SP12: *Myrmeleotettix palpalis*; SP13: *Oedipoda caerulelescens*; SP14: *Dericorys tibialis*; SP15: *Sphingonotus coerulipes*; SP16: *Sphingonontus salinus*; SP17: *Omocestus viridulus*; SP18: *Bryodema mongolicum*; SP19: *Omocestus Petraeus*; SP20: *Stenobothrus lineatus*; SP21: *Ramburiella turcomana*; SP22: *Pyrgodera armata*; SP23: *Calliptamus coelesyriensis*; SP24: *Aeropus sibiricus*; SP25: *Pararcyptera microptera microptera*; SP26: *Notostaurus albicornis*; SP27: *Helioscirtus moseri moseri*; SP28: *Sphingonolus nebulosus nebulosus*; SP29: *Chorthippus biguttulus*; SP30: *Calliptamus italicus*; SP31: *Egnatius apicalis*; SP32: *Conophyma zhaosuensis*; SP33: *Stauroderus scalaris scalaris*; SP34: *Bryodema gebleri*.

Figure 5 depicts the niche breadth of the locust species across the different grassland types. Overall, *Dociostaurus* spp. exhibited the largest niche breadth (31.64), although this value represents a pooled estimate for multiple morphologically similar species within the genus that could not be rapidly distinguished under field conditions. In contrast, several species showed the smallest niche breadth (1.00), including *Parapleurus alliaceus*, *Euchorthippus pulvinatus* (Fischer von Waldheim, 1846), *Chorthippus dichrous* (Eversmann, 1859), *Myrmeleotettix palpalis* (Zubovski, 1900), *Egnatius apicalis* (Stål, 1876), and *Conophyma zhaosuensis* Huang, 1982. The niche breadth of the remaining species ranged between 1.25 and 20.88.

**Figure 6:**
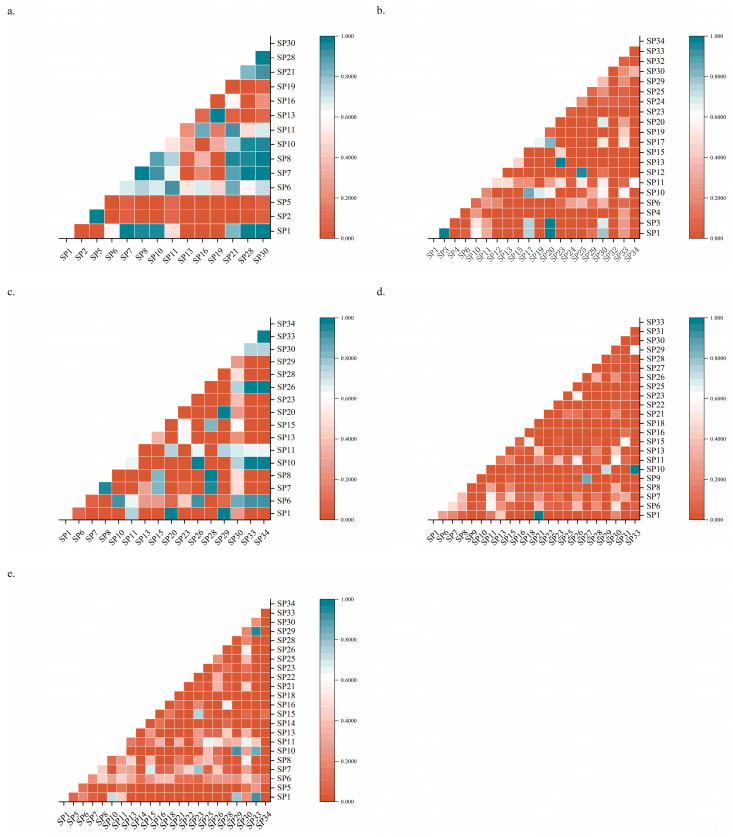
Niche overlap semi-matrix of locust species across grassland types in the Sino-Kazakh border region. Note: (**a**): lowland meadows, (**b**): mountain meadows, (**c**): temperate steppes, (**d**): temperate desert-steppes, (**e**): temperate desert grasslands. The explanation of the SP number is the same as that in Figure 5.

In the lowland meadows, *Oedaleus decorus decorus* showed the greatest niche breadth (2.92), whereas *Chorthippus albomarginatus*, *Sphingonotus nebulosus nebulosus*, *Sphingonotus salinus* (Pallas, 1773), *Celes variabilis variabilis* (Pallas, 1771), *Omocestus petraeus,* and *Parapleurus alliaceus* had the smallest niche breadth (1.00). Other species exhibited niche breadth values between 1.20 and 2.68. Among the 91 species pairs, 35 pairs (38.64%) had niche overlap values > 0.6 (see Figure 6a).

In the mountain meadows, *Stauroderus scalaris scalaris* had the highest niche breadth (8.67), while the lowest values (1.00) were observed in *Oedipoda caerulelescen*, *Omocestus petraeus*, *Bryodema gebleri*, *Euchorthippus pulvinatus*, *Chorthippus dichrous*, *Myrmeleotettix palpalis*, and *Conophyma zhaosuensis*. The remaining species had niche breadth values ranging from 1.20 to 5.29. Out of 190 species pairs, 15 (7.89%) demonstrated a niche overlap > 0.6 (see Figure 6b).

Within the temperate steppes, *Calliptamus italicus* showed the largest niche breadth (3.46), and the smallest niche breadths (1.00) were recorded for *Stauroderus scalaris scalaris*, *Omocestus haemorrhoidalis*, *Chorthippus biguttulus*, *Chorthippus albomarginatus*, *Calliptamus coelesyriensis*, *Stenobothrus lineatus*, *Bryodema gebleri*, *Oedipoda miniata*, *Sphingonotus nebulosus nebulosus*, and *Notostaurus albicornis*. Other species displayed a niche breadth between 1.96 and 2.16. Of the 120 species pairs, 34 (28.33%) exhibited niche overlap values > 0.6 (see Figure 6c).

In the temperate desert-steppes, *Calliptamus italicus* had the highest niche breadth (11.81), with the lowest (1.00) values recorded for *Sphingonotus salinus*, *Chorthippus biguttulus*, *Pyrgodera armata* (Fischer von Waldheim, 1820), *Bryodema mongolicum* (Fischer von Waldheim, 1836), and *Celes variabilis variabilis*. The niche breadth of the other species varied from 1.60 to 10.53. Among the 231 species pairs, 7 (3.03%) displayed a niche overlap value > 0.6 (see Figure 6d).

In the temperate desert grasslands, *Dociostaurus* spp. possessed the greatest niche breadth (14.90), while *Chorthippus biguttulus*, *Chorthippus albomarginatus*, *Pararcyptera microptera microptera*, *Pyrgodera armata*, *Bryodema mongolicum*, and *Eganatius apicalis* showed the smallest niche breadth (1.00). The remaining species had niche breadth values between 1.46 and 12.23. Out of the 231 species pairs, 12 (5.19%) demonstrated a niche overlap value > 0.6 (see Figure 6e).

### 3.5. Locust Response to Environmental Factors in the Sino-Kazakh Border Region

Initial VIF analysis identified three variables with extremely high collinearity (VIF > 100): soil salinity (VIF = 105.692), soil electrical conductivity (VIF = 117.196), and total dissolved solids (VIF = 127.968). These were excluded from further analyses. After removal, VIF values for all remaining environmental variables ranged from 3.21 to 8.91, all below the threshold of 10 (Table S2), indicating that multicollinearity was effectively addressed. The PCA indices revealed that PC1 explained 11.3% of the total variance. The loadings of the original variables on PC1 are presented in Supplementary Table S3.

**Figure 7:**
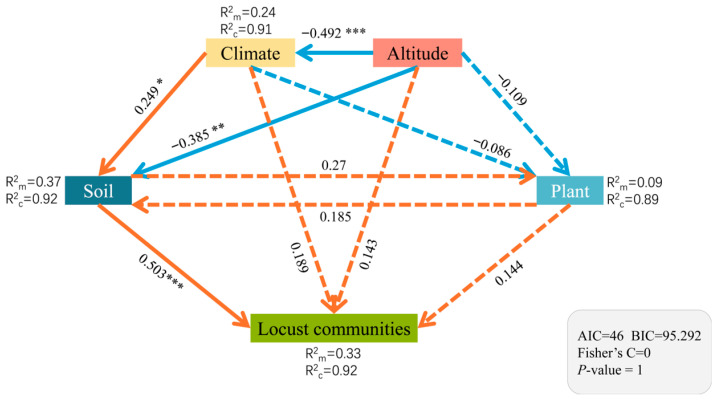
Piecewise Structural Equation Modeling (PiecewiseSEM). Note: Orange arrows indicate significant positive paths; blue arrows represent significant negative paths; orange dashed lines denote non-significant positive paths; blue dashed lines indicate non-significant negative paths. Numbers adjacent to arrows represent standardized path coefficients. Marginal R^2^ (R^2^m) and conditional R^2^ (R^2^c) denote the proportion of variance explained by all predictors without and with the inclusion of random effects, respectively. AIC: Akaike’s Information Criterion; BIC: Bayesian Information Criterion; Fisher’s C: Fisher’s combined probability test. Asterisks indicate significance levels: * *p* < 0.05, ** *p* < 0.01, *** *p* < 0.001.

**Figure 8:**
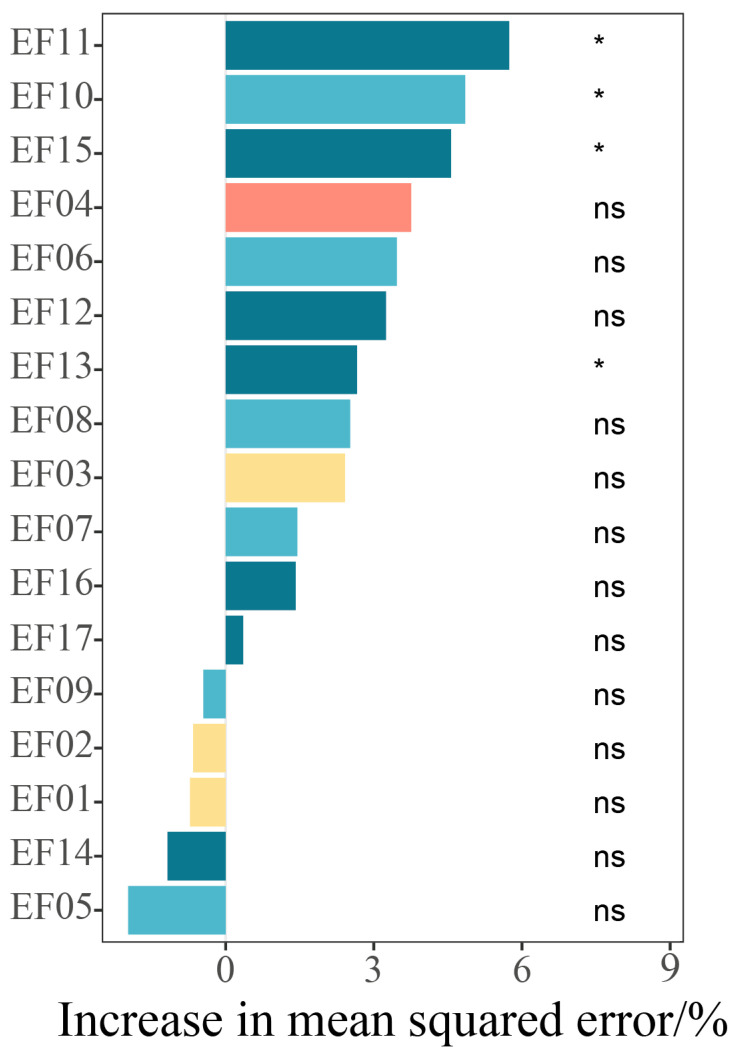
Analysis of the Random Forest (RF) Model. Note: EF01: mean annual soil temperature; EF02: mean annual wind speed; EF03: mean annual precipitation; EF04: elevation; EF05: vegetation height; EF06: vegetation coverage; EF07: plant organic carbon content; EF08: plant total nitrogen content; EF09: plant total phosphorus content; EF10: plant total potassium content; EF11: soil organic carbon (SOC) content; EF12: soil total nitrogen content; EF13: soil total phosphorus content; EF14: soil total potassium content; EF15: soil pH; EF16: soil bulk density; EF17: soil water content (%). Asterisk (*) indicates significance at *p* < 0.05; “ns” denotes non-significance.

The results of the PiecewiseSEM analyzing the direct and indirect effects of key environmental factors on locust communities demonstrated a good model fit (Fisher’s C = 0, *p* = 1, AIC = 46, BIC = 95.292) and are presented in Figure 7. The soil variables exerted a highly significant direct effect on the locust communities (*p* < 0.001). The conditional R^2^ values for the endogenous variables were soil properties (R^2^c = 0.92), climate (R^2^c = 0.91), altitude (R^2^c = 0.27), plant characteristics (R^2^c = 0.89), and locust communities (R^2^c = 0.92). In contrast, elevation and climatic factors collectively had no significant direct impact, but they indirectly influenced the locust communities by modulating the soil properties. The RF analysis (Figure 8) further identified the SOC, soil pH, soil TP, and plant total potassium as significant predictors of locust communities (*p* < 0.05). These variables explained 5.8%, 4.6%, 2.8%, and 4.8% of the variation in locust density, respectively.

## 4. Discussion

Grassland types differ substantially in their capacity to provide resources for grasshopper development, resulting in distinct patterns of species richness and distribution across habitats [40]. Current results revealed that *Dociostaurus* spp., *Calliptamus italicus*, and *Calliptamus barbarus barbarus* were dominant in Xinjiang along the Sino-Kazakh border. This dominance demonstrated a pattern of large niche breadth coupled with low niche overlap. Despite their broad ecological niche breadths—indicating extensive utilization potential across diverse environmental resources—these species had consistently low niche overlap indices (<0.6) with sympatric taxa. This suggests that these dominant species do not use generalist competitive strategies but rather occupy space via multidimensional resource partitioning that enables stable coexistence. Such niche differentiation substantially reduces potential competition and can result in an equilibrium between resource-use efficiency and competitive release [41]. These findings show that widespread species can maintain broad niches without competitive exclusion and improve current understanding of insect community assembly and biodiversity maintenance in arid and semi-arid grasslands. Similar patterns of dominant species distribution across heterogeneous grassland habitats have been documented in European calcareous grasslands, where community composition is strongly influenced by local habitat conditions [42].

The narrow niche breadth observed in some species (e.g., *Conophyma zhaosuensis* and *Myrmeleotettix palpalis*) suggests they may have specific habitat requirements. This pattern aligns with patterns expected under niche conservatism [43,44], although testing this explicitly would require phylogenetic data not covered in this study. Studies on Andean grasshoppers have similarly documented that closely related species tend to retain similar ecological niches over evolutionary time, contributing to patterns of local endemism [45].

Although *Dociostaurus* spp. and *Calliptamus italicus* were widely distributed across the Sino-Kazakh border region, the dominant species within the locust communities differed significantly between the Kazakh and Chinese (Xinjiang) sides. Typical communities on the Kazakh side were dominated by *Locusta migratoria*, *Dociostaurus maroccanus* (Thunberg, 1815), and *Calliptamus italicus* [46], whereas the Xinjiang section was dominated by *Calliptamus italicus*, *Calliptamus barbarus barbarus*, and *Dociostaurus tartarus* Stshelkanovtzev, 1921. *Locusta migratoria* prefers reed habitats with high moisture availability [47], while *Calliptamus italicus* tends to inhabit relatively arid temperate desert steppes [48]. The Alakol locust area in Kazakhstan, which features specific vegetation, a low soil pH, reduced salinity, moderate organic matter content, and suitable precipitation, provides optimal conditions for *Locusta migratoria*, supporting its dominance in these communities. In contrast, Liu et al. reported significantly higher concentrations of Cl^−^, SO_4_^2−^, K^+^, Na^+^, Mg^2+^, and HCO_3_^−^ in the soils of the Tacheng border region in China compared with the Alakol area in Kazakhstan [49]. Furthermore, extensive tracts of temperate desert steppe and abandoned farmland on the Chinese side of the border provide ideal breeding conditions for *Calliptamus italicus* and *Calliptamus barbarus barbarus*. This habitat heterogeneity likely drives pronounced spatial divergence in the grasshopper community structure. The realized niches of widespread species, while broadly similar at the regional scale, show local-scale variation in both niche breadth and niche overlap corresponding to differences in environmental conditions. Specifically, the degree of niche overlap among co-occurring species exhibited systematic variation across grassland types (Figure 6), and these patterns appeared to be closely associated with local-scale environmental factors such as soil properties and vegetation characteristics. This context-dependency of niche relationships is consistent with hierarchical models of community assembly, which propose that species distributions are shaped by the combined effects of species traits, biotic interactions, and environmental filtering [50,51].

Here, contrasting patterns between species richness and niche overlap across grassland types were observed. The warm temperate desert-steppe and warm temperate desert grassland had the highest number of recorded locust species (22 species each), which was accompanied by the lowest proportions of species pairs with high niche overlap (only 3.03% and 5.19% of species pairs, respectively, had overlap values > 0.6; Figure 6d,e). Conversely, the lowland meadow had a relatively low number of locust species (14 species) but the highest proportion of species pairs with high niche overlap (38.64% of species pairs demonstrated overlap > 0.6). There are several potential explanations for this. Firstly, although the warm temperate desert types have relatively low primary productivity, they are widely distributed and characterized by distinct patchiness (such as bare land, shrub patches, and various herbaceous patches), creating highly heterogeneous microhabitats. This provides diverse niche opportunities for a larger number of species. In contrast, the lowland meadow has a humid habitat with dense vegetation and relatively homogeneous conditions. Despite its high primary productivity, the resource diversity (such as plant species and microtopography) is low. This forces species to concentrate on a few high-quality resources, significantly increasing niche overlap and triggering intense interspecific competition. Under competitive exclusion, only a few dominant species with strong competitiveness (such as *Calliptamus italicus*) can survive and dominate. This ultimately leads to a relatively small total number of species with high competition intensity.

Secondly, disturbances, such as grazing in desert ecosystems, are often more random, which helps to maintain habitat heterogeneity and prevents the dominance of one species. In contrast, lowland meadows are often used as high-quality pastures. This frequent and stable disturbance may simplify the environment and intensify competition. Thirdly, in arid and semi-arid desert environments, abiotic stresses (such as water scarcity and temperature fluctuations) are the main limiting factors. Under such conditions, the population density of the locusts is intensely restricted by resources (such as food and oviposition sites). Consequently, environmental stress suppresses the competition intensity and allows for more species to coexist. Therefore, higher habitat heterogeneity supports greater species diversity and weakens competition by promoting niche differentiation [52], while habitats with abundant resources but simple structures lead to high niche overlap, intense competition, and reduced species richness [53]. This supports the essential differences in community assembly mechanisms among different grassland ecosystems.

In this study, the locust community assembly was shaped by multi-level environmental drivers through complex pathways. Large-scale geographic and climatic factors, such as elevation, mean annual temperature, and precipitation, did not exhibit significant direct effects on the locust communities. However, they exerted highly significant indirect effects (*p* < 0.01) by mediating pedogenic processes, influencing key soil properties, including temperature, moisture, and nutrient cycling. These findings indicated that macro-environmental drivers affect locust communities primarily through soil-mediated pathways rather than through direct biological effects [54]. In contrast, soil variables, including the organic matter content, key nutrients (e.g., nitrogen and phosphorus), soil texture, and pH, showed strong direct effects (*p* < 0.001). This underscores the central role of soil properties in linking macro-environmental conditions and biological responses [55]. For instance, climatic aridification may indirectly favor locust species with enhanced drought tolerance and egg diapause, such as *Calliptamus italicus*, by reducing the soil organic matter and intensifying moisture stress. This provides a plausible mechanism that could explain the gradual replacement of *Locusta migratoria* by *Calliptamus italicus* as the dominant species in the grassland ecosystems of Xinjiang.

The RF model further quantified the heterogeneity of the environmental driving forces, where the key soil factors contributing to variation in the locust community were the soil organic carbon content, pH value, total phosphorus content, and plant total potassium content (*p* < 0.05). Their combined effects regulate the synthesis of plant nutrients and secondary metabolites, thereby altering the quality and availability of food resources for locusts [56,57,58]. This finding is supported by Li et al. [40], who found that locust diversity is negatively correlated with soil pH and positively correlated with moisture conditions. Thus, moisture conditions are likely to indirectly regulate the physical and chemical properties of the soil, such as the rate of organic matter mineralization and the pH dynamics. Additionally, Ji et al. [59] showed that soil moisture content and salinity influence the oviposition of migratory locusts. This supports the crucial role of soil factors in the establishment of locust populations.

In contrast to some previous studies [41,60,61], current analyses did not identify any significant independent effects of vegetation factors (diversity, coverage, and height) on locust community structure after accounting for soil properties. This discrepancy likely stems from methodological differences in variable selection. For example, whereas prior studies focused predominantly on the isolated effects of plant factors, this study systematically evaluated the contribution of the soil properties. It was demonstrated that vegetation characteristics (including diversity, biomass, and spatial configuration) were not fully independent explanatory variables but rather response variables shaped by the underlying soil conditions (nutrient availability, pH, and moisture status) [62]. Consequently, previously reported vegetation–locust correlations may have reflected indirect soil effects rather than direct causal relationships. Nevertheless, plant factors retain ecological relevance. The RF modeling identified plant total potassium content as a significant predictor of locust community structure. This indicates that the soil attributes indirectly influence locusts through the vegetation, with effects on the plant structural and functional traits.

Finally, it is acknowledged that sweep-net sampling, while widely used and standardized in this study, may have inherent biases (e.g., toward larger-bodied or more active species) that could influence abundance estimates and should be considered when interpreting results. It should also be noted that the number of sampling plots (i.e., resource states, N) varied among grassland types, which may affect the comparability of raw niche breadth values across habitats. Future studies with more balanced sampling designs would allow for more robust cross-habitat comparisons. Spatial autocorrelation was not explicitly modelled in this study, and the potential influence of spatial structure on the observed patterns cannot be entirely ignored. In summary, the assembly of locust communities in arid and semi-arid ecosystems is co-regulated by a hierarchical framework of three primary mechanisms: niche differentiation (reducing potential interspecific competition), niche constraints (limiting specialist species), and environmental filtering (modulating community composition through abiotic factors). This integrated framework offers a robust theoretical basis for predicting community responses to global change. The structure of locust communities in the China–Kazakhstan border region is a result of macro-climatic patterns that are filtered and modulated through soil properties. These climatic factors exert strong indirect effects by altering the soil physicochemical properties that shape habitat conditions and resource availability for locusts. These findings enhance our understanding of cross-scale “climate–soil–biota” cascading ecological processes. They also propose a new direction for regional locust management through shifting from direct population control toward sustainable soil improvement and vegetation restoration. By targeting the underlying habitat suitability through ecological remediation, more effective and long-term pest governance could be achieved.

## Figures and Tables

**Table 1:** Dominant locust species and their relative dominance across different grassland types in the Xinjiang region along the Sino-Kazakh border.

Species Code	Species Name	Family Name	Genus Name	Lowland Meadows	Mountain Meadows	Temperate Steppes	Temperate Desert-Steppes	Temperate Desert Grasslands
SP1	*Chorthippus albomarginatus*	Arcypteridae	*Chorthippus*	0.35%	3.61%	1.01%	0.10%	1.95%
SP2	*Parapleurus alliaceus*	Oedipodidae	*Parapleurus*	7.80%				
SP3	*Euchorthippus pulvinatus*	Arcypteridae	*Euchorthippus*		0.93%			
SP4	*Chorthippus dichrous*	Arcypteridae	*Chorthippus*		0.12%			
SP5	*Celes variabilis variabilis*	Oedipodidae	*Celes*	0.71%				0.06%
SP6	*Oedaleus decorus decorus*	Oedipodidae	*Oedaleus*	10.64%	1.28%	19.70%	9.59%	6.53%
SP7	*Calliptamus barbarus barbarus*	Catantopidae	*Calliptamus*	7.45%		18.18%	15.80%	19.81%
SP8	*Oedipoda miniata*	Oedipodidae	*Oedipoda*	6.74%		1.01%	9.29%	4.12%
SP9	*Dericorys annulata roseipennis*	Catantopidae	*Dericorys*				0.20%	
SP10	*Omocestus haemorrhoidalis*	Arcypteridae	*Omocestus*	1.42%	5.47%	1.01%	0.07%	2.92%
SP11	*Dociostaurus spp.*	Arcypteridae	*Dociostaurus*	19.86%	37.72%	21.72%	21.32%	24.33%
SP12	*Myrmeleotettix palpalis*	Gomphoceridae	*Myrmeleotettix*		0.47%			
SP13	*Oedipoda caerulelescens*	Oedipodidae	*Oedipoda*	3.90%	0.23%	3.54%	0.47%	1.26%
SP14	*Dericorys tibialis*	Catantopidae	*Dericorys*					0.69%
SP15	*Sphingonotus coerulipes*	Oedipodidae	*Sphingonotus*		0.35%	3.54%	2.20%	0.86%
SP16	*Sphingonontus salinus*	Oedipodidae	*Sphingonotus*	0.71%			0.37%	0.11%
SP17	*Omocestus viridulus*	Arcypteridae	*Omocestus*		1.63%			
SP18	*Bryodema mongolicum*	Oedipodidae	*Bryodema*				0.03%	0.46%
SP19	*Omocestus petraeus*	Arcypteridae	*Omocestus*	1.06%	0.12%			
SP20	*Stenobothrus lineatus*	Arcypteridae	*Stenobothrus*		1.28%	1.01%		
SP21	*Ramburiella turcomana*	Arcypteridae	*Ramburiella*	16.67%			0.54%	0.92%
SP22	*Pyrgodera armata*	Oedipodidae	*Pyrgodera*				0.03%	0.11%
SP23	*Calliptamus coelesyriensis*	Catantopidae	*Calliptamus*		2.33%	0.51%	12.61%	3.15%
SP24	*Aeropus sibiricus*	Gomphoceridae	*Aeropus*		4.07%			
SP25	*Pararcyptera microptera microptera*	Arcypteridae	*Pararcyptera*		4.31%		0.27%	0.34%
SP26	*Notostaurus albicornis*	Arcypteridae	*Notostaurus*			1.01%	1.29%	0.80%
SP27	*Helioscirtus moseri moseri*	Oedipodidae	*Helioscirtus*				0.14%	
SP28	*Sphingonolus nebulosus nebulosus*	Oedipodidae	*Sphingonotus*	0.71%		5.56%	0.10%	0.97%
SP29	*Chorthippus biguttulus*	Arcypteridae	*Chorthippus*		5.36%	5.05%	0.03%	0.86%
SP30	*Calliptamus italicus*	Catantopidae	*Calliptamus*	21.99%	13.39%	7.58%	25.15%	28.73%
SP31	*Egnatius apicalis*	Gomphoceridae	*Egnatius*				0.07%	
SP32	*Conophyma zhaosuensis*	Catantopidae	*Conophyma*		0.70%			
SP33	*Stauroderus scalaris scalaris*	Arcypteridae	*Stauroderus*		13.97%	0.51%	0.31%	0.34%
SP34	*Bryodema gebleri*	Oedipodidae	*Bryodema*		2.68%	9.09%		0.69%

**Table 2:** Analysis of the Similarity of Locust Communities in Different Grassland Types in the Sino-Kazakh Border Region.

	Lowland Meadows	Mountain Meadows	Temperate Steppes	Temperate Desert-Steppes	Temperate Desert Grasslands
Lowland meadows	1.00	0.14	0.34	0.07	0.12
Mountain meadows	0.14	1.00	0.12	0.15	0.29
Temperate steppes	0.34	0.12	1.00	0.05	0.11
temperate desert-steppes	0.07	0.15	0.05	1.00	0.52
Temperate desert grasslands	0.12	0.29	0.11	0.52	1.00

Note: Values in the table below 0.15 indicate low similarity, 0.15–0.40 indicate moderate similarity, and above 0.4 indicate high similarity.

## Data Availability

The original contributions presented in this study are included in the article/Supplementary Material. Further inquiries can be directed to the corresponding author.

## Supplementary Materials

The following supporting information can be downloaded at: https://www.mdpi.com/article/10.3390/insects17030348/s1, Figure S1: 3D NMDS Ordination plot of Grasshopper Communities in Different Grassland Types in the Sino-Kazakh Border Region. Table S1: The Raw Species Abundance Matrix for Each Sampling Plot. Table S2: Variance inflation factor (VIF) for environmental variables before and after excluding highly collinear variables. Table S3: Loadings of grasshopper species on the first two principal components (PC1 and PC2) from principal component analysis based on species abundance data. Table S4: Environmental variables (soil properties and vegetation characteristics) for each plot.
