# Supplementary material for: Spatial Differentiation and Environment-Driven Mechanisms of Locust Community Structure in the Xinjiang Region Along the Sino-Kazakh Border
Source: Insects. 2026 Mar 22;17(3):348. doi: 10.3390/insects17030348 (PMC13026512; doi:10.3390/insects17030348)
Supplement: Supplementary file 1 [file insects-17-00348-s001.zip › table S2.pdf]

Table S2. Variance inflation factor (VIF) for environmental variables before and after excluding highly collinear variables.

Variable	Initial VIF	Action	Final VIF (after exclusion)
Mean annual soil temperature	4.437	Retained	4.361
Mean annual wind speed	1.583	Retained	1.564
Mean annualprecipitation	2.818	Retained	2.627
Elevation	6.945	Retained	6.588
Vegetation height	2.044	Retained	1.920
Vegetation coverage	2.434	Retained	2.185
Plant or-ganic carbon-content	1.480	Retained	1.446
Plant total nitrogen content	3.047	Retained	2.742
Plant total phosphorus content;	3.427	Retained	3.055
Plant total potassium content	1.827	Retained	1.629
Soil organic carbon content	12.187	Retained	9.442
Soil total nitrogen content	4.190	Retained	3.715
Soil total phosphorus content	1.681	Retained	1.561
Soil total potassium-content	2.096	Retained	1.983
Soil pH	3.056	Retained	2.870
Soil salinity	105.692	Removed	——
Soil electrical conductivity	117.196	Removed	——
Total dissolved solids	127.968	Removed	——
Soil bulk density	4.743	Retained	3.015
Soil water content	5.379	Retained	4.671
