# Supplementary material for: Spatial Differentiation and Environment-Driven Mechanisms of Locust Community Structure in the Xinjiang Region Along the Sino-Kazakh Border
Source: Insects. 2026 Mar 22;17(3):348. doi: 10.3390/insects17030348 (PMC13026512; doi:10.3390/insects17030348)
Supplement: Supplementary file 1 [file insects-17-00348-s001.zip › table S3.pdf]

Table S3. Loadings of grasshopper species on the first two principal components (PC1 and PC2) from principal component analysis based on species abundance data

Species code	**Species name**	PC1 loading	PC2 loading
SP1	*Chorthippus albomarginatus*	0.432	-0.066
SP2	*Parapleurus alliaceus*	-0.018	0.148
SP3	*Euchorthippus pulvinatus*	0.445	-0.049
SP4	*Chorthippus dichrous*	0.030	0.100
SP5	*Celes variabilis variabilis*	-0.017	0.151
SP6	*Oedaleus decorus decorus*	-0.057	-0.373
SP7	*Calliptamus barbarus barbarus*	-0.090	-0.252
SP8	*Oedipoda miniata*	-0.052	-0.235
SP9	*Dericorys annulata roseipennis*	-0.032	0.227
SP10	*Omocestus haemorrhoidalis*	0.319	-0.031
SP11	*Dociostaurus spp.*	0.082	-0.441
SP12	*Myrmeleotettix palpalis*	-0.002	-0.088
SP13	*Oedipoda caerulelescens*	-0.045	-0.048
SP14	*Dericorys tibialis*	-0.018	0.062
SP15	*Sphingonotus coerulipes*	-0.078	0.015
SP16	*Sphingonontus salinus*	-0.028	0.024
SP17	*Omocestus viridulus*	0.433	0.022
SP18	*Bryodema mongolicum*	-0.023	0.022
SP19	*Omocestus petraeus*	0.039	0.050
SP20	*Stenobothrus lineatus*	0.455	-0.037
SP21	*Ramburiella turcomana*	-0.023	-0.164
SP22	*Pyrgodera armata*	-0.032	-0.053
SP23	*Calliptamus coelesyriensis*	-0.041	-0.144
SP24	*Aeropus sibiricus*	-0.014	0.078
SP25	*Pararcyptera microptera microptera*	0.005	-0.077
SP26	*Notostaurus albicornis*	-0.041	-0.334
SP27	*Helioscirtus moseri moseri*	-0.027	0.211
SP28	*Sphingonolus nebulosus nebulosus*	-0.035	-0.030
SP29	*Chorthippus biguttulus*	0.119	0.047
SP30	*Calliptamus italicus*	0.039	-0.382
SP31	*Egnatius apicalis*	-0.036	0.012
SP32	*Conophyma zhaosuensis*	-0.009	0.073
SP33	*Stauroderus scalaris scalaris*	0.244	0.191
SP34	*Bryodema gebleri*	-0.011	-0.070
