# Supplementary figures and images for: Spatial Differentiation and Environment-Driven Mechanisms of Locust Community Structure in the Xinjiang Region Along the Sino-Kazakh Border
Source: Insects. 2026 Mar 22;17(3):348. doi: 10.3390/insects17030348 (PMC13026512; doi:10.3390/insects17030348)

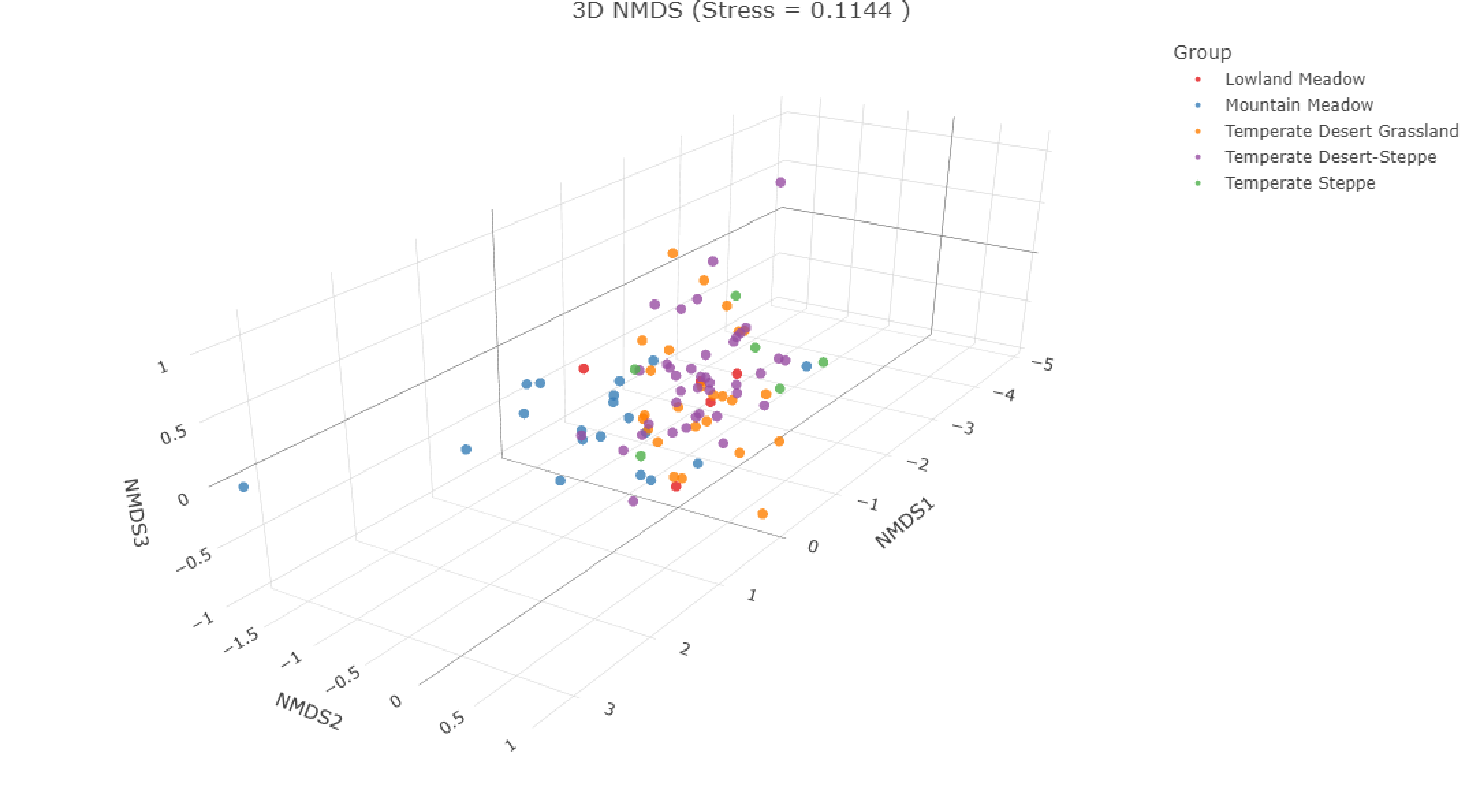

Supplement: Supplementary file 1 [file insects-17-00348-s001.zip › Figure S1.tif]
